# Itaconate promotes a wound resolving phenotype in pro-inflammatory macrophages

**DOI:** 10.1016/j.redox.2022.102591

**Published:** 2022-12-24

**Authors:** Sjors Maassen, Britt Coenen, Melina Ioannidis, Karl Harber, Pieter Grijpstra, Jan Van den Bossche, Geert van den Bogaart

**Affiliations:** a Department of Molecular Immunology, Groningen Biomolecular Sciences and Biotechnology, University of Groningen, Groningen, the Netherlands; b Department of Molecular Cell Biology and Immunology, Amsterdam Cardiovascular Sciences, Amsterdam Gastroenterology Endocrinology Metabolism, Amsterdam Institute for Infection and Immunity, Cancer Centre Amsterdam, Amsterdam UMC, Vrije Universiteit Amsterdam, Amsterdam, the Netherlands; c Department of Medical Biology and Pathology, University Medical Center Groningen, Groningen, the Netherlands

## Abstract

Pathological conditions associated with dysfunctional wound healing are characterized by impaired remodelling of extracellular matrix (ECM), increased macrophage infiltration, and chronic inflammation. Macrophages also play an important role in wound healing as they drive wound closure by secretion of molecules like transforming growth factor beta-1 (TGF-β). As the functions of macrophages are regulated by their metabolism, local administration of small molecules that alter this might be a novel approach for treatment of wound-healing disorders. Itaconate is a tricarboxylic acid (TCA) cycle-derived metabolite that has been associated with resolution of macrophage-mediated inflammation. However, its effects on macrophage wound healing functions are unknown. In this study, we investigated the effects of the membrane-permeable 4-octyl itaconate (4-OI) derivative on ECM scavenging by cultured human blood monocyte-derived macrophages (hMDM). We found that 4-OI reduced signalling of p38 mitogen-activated protein kinase (MAPK) induced by the canonical immune stimulus lipopolysaccharide (LPS). Likely as a consequence of this, the production of the inflammatory mediators like tumor necrosis factor (TNF)-α and cyclooxygenase (COX)-2 were also reduced. On the transcriptional level, 4-OI increased expression of the gene coding for TGF-β (*TGFB1*), whereas expression of the collagenase matrix metalloprotease-8 (*MMP8*) was reduced. Furthermore, surface levels of the anti-inflammatory marker CD36, but not CD206 and CD11c, were increased in these cells. To directly investigate the effect of 4-OI on scavenging of ECM by macrophages, we developed an assay to measure uptake of fibrous collagen. We observed that LPS promoted collagen uptake and that this was reversed by 4-OI-induced signaling of nuclear factor erythroid 2–related factor 2 (NRF2), a regulator of cellular resistance to oxidative stress and the reduced glycolytic capacity of the macrophage. These results indicate that 4-OI lowers macrophage inflammation, likely promoting a more wound-resolving phenotype.

## Graphical abstract

**Visual abstract.** Inflammatory macrophages stimulated by Toll-like receptor 4 (TLR4) signaling efficiently ingest collagen by gactivation of p38 signaling. Itaconate inhibits p38 signaling and promotes NRF2 signaling, thereby reducing collagen uptake. Itaconate also inhibits expression of TNF-α and COX2.

**Figure:**
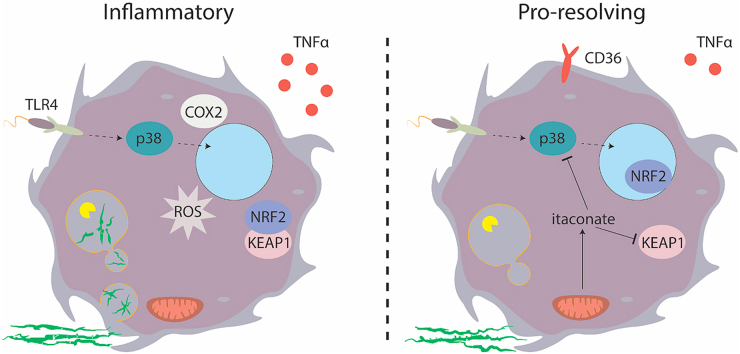

## 1 Introduction

Diseases and disorders associated with impaired wound healing, such as type II diabetes and vascular disease, are a significant burden on the quality of life for patients and have a substantial societal and economic impact on health care [1]. Wound healing is also thought to decline with age [2]. One of the prevalent phenotypes of impaired wound healing is prolonged inflammation associated with increased infiltration of macrophages into the damaged tissue [3]. The macrophage is a versatile immune cell type that plays large roles in wound healing. Following the damage of tissue, macrophages activate inflammatory pathways to destroy pathogens by phagocytosis and cytotoxicity mechanisms and secrete inflammatory chemokines and cytokines to control infections (*e.g.,* tumour necrosis factor-alpha (TNF-α)). However, once the infection is cleared, macrophages also mediate the clearance of tissues damaged from fighting the infection [4], excessively produced extracellular matrix (ECM), and cell debris; an important feature in the resolution of inflammatory responses. This macrophage phenotype is regarded as a pro-resolution macrophage that drives wound healing, for instance by secreting transforming growth factor beta (TGF-β). Insufficient clearance of ECM contributes to scarring and fibrosis, whereas the overactivation of this process might lead to impaired wound healing. The clearance of ECM by macrophages is also important in embryonic and tissue development.

Macrophages regulate their function by adapting their metabolism [5,6]. For instance, itaconate, a metabolite produced in the Krebs cycle by immune responsive gene 1 (IRG1; also known as aconitate decarboxylase 1, ACOD1) covalently binds to cysteines in the proteome of macrophages [7,8]. This covalent modification also affects micro-organisms exposed to itaconate, thereby limiting their growth and making itaconate an anti-microbial compound [9]. During the later stages of inflammation, alkylation by itaconate (and cell-permeable derivatives) in the macrophage activates the nuclear factor-erythroid factor 2-related factor 2 (NRF2) signalling pathway by the inhibition of Kelch-like ECH-associated protein 1 (KEAP1) [7], resulting in the increased detoxification of reactive oxygen species (ROS) and inhibition of transcription of genes coding for inflammatory cytokines (IL-6, IL-1β, IFN-β) [10,11]. Moreover, itaconate has been shown to bind activating transcription factor 3 (ATF3), which activates anti-inflammatory pathways in the macrophage [12]. These processes promote the resolution of inflammation and contribute to homeostasis (*e.g.,* by alleviating fibrosis) [13]. Macrophages highly expressing IRG1 are present in wounds of mice after four days of wounding [14]. In the early phase of inflammation itaconate alkylates succinate dehydrogenase causing a break in the TCA cycle, which leads to the accumulation of succinate [15]. Succinate production in turn leads to the stabilisation of HIF-1α in macrophages, contributing to the pro-inflammatory response [16]. Time-resolved single cell RNA-sequencing has shown that this HIF-1α response determines the progression of wound-resident macrophages into a wound healing phenotype [17]. These findings suggest that itaconate might also contribute to a well-known transformation of macrophages from an inflammatory to a pro-resolution phenotype in wounds and inflamed tissue [18]. However, how itaconate contributes to wound healing by modulating macrophages is not yet understood.

In this study, we investigated the role of IRG1 and 4-octyl itaconate (4-OI), a cell-permeable derivative of itaconate, in type-I collagen scavenging, an essential process in wound healing, in human peripheral blood monocyte-derived macrophages (hMDM). hMDMs are increased in wounds during healing [19] and are an important immune cell type that plays key roles during all stages of wound healing [20] making them a relevant model to study *in vitro* in this context. We show that 4-OI reduces the secretion of pro-inflammatory cytokine TNF-α induced by the pathogenic stimulus lipopolysaccharide (LPS). In accordance with this, the knockdown of IRG1 increases TNF-α production. In addition, we show that 4-OI specifically reduces the uptake of fibrous type I collagen, but not of other endocytic and phagocytic cargoes, likely due to the lower expression of scavenger receptors on the macrophage surface. 4-OI has also been shown to reduce the glycolysis in murine macrophages [21], which we demonstrate to be an important metabolic feature that reduces the ingestion of collagen. Our data suggest that 4-OI impairs the metabolism of the hMDMs in both lipid to glucose uptake, of which the latter also affects collagen uptake. The inhibition of collagen uptake by 4-OI was also caused by its induction of NRF2 signalling, as we found that collagen uptake could be rescued by inhibition of NRF2. 4-OI also inhibited activation of p38 mitogen-activated protein kinase (MAPK), and inhibition of p38 reduced LPS-induced collagen uptake. Furthermore, these results indicate that 4-OI could be used for treatment of diseases and disorders associated with excessive collagen scavenging.

## 2 Results

### 2.1 Pro-inflammatory macrophages increase type I collagen uptake

**Fig. 1:**
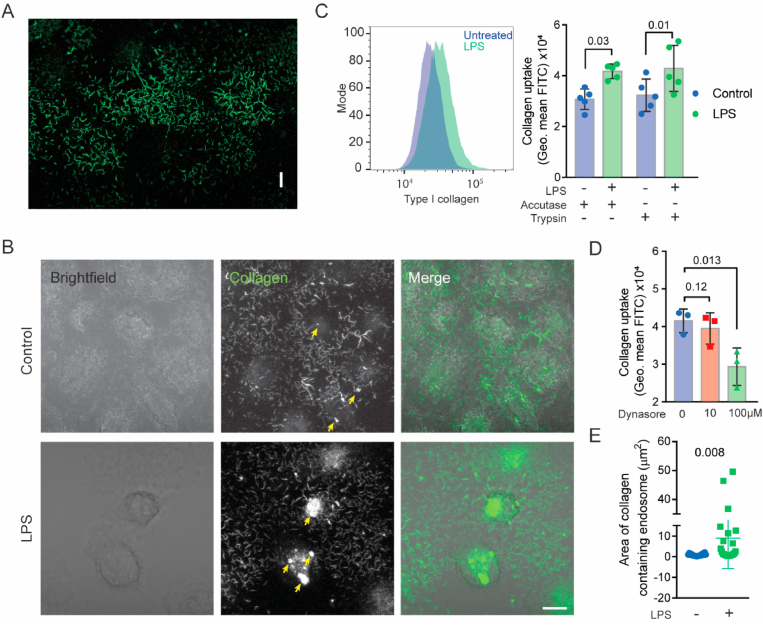
**LPS promotes collagen uptake and intracellular degradation by human blood monocyte derived macrophages (hMDM). A)** Fluorescence microscopy of fibrous collagen in a cell culture dish. Collagen was labelled with FITC. Scalebar is 10 μm. **B)***Z*-projection of live-cell confocal micrographs of macrophages seeded on fibrous FITC-collagen for 24 h. Note the large intracellular compartments (yellow arrows) containing collagen in LPS-stimulated macrophages (24 h stimulation) compared to control. Scalebar is 10 μm. **C)** Representative flow cytometry histogram and quantification of macrophages cultured on FITC-labelled collagen with and without LPS for 24 h, and subsequently detached with either Accutase or Trypsin (n = 5 donors, one-way ANOVA with Tukey's multiple comparison test). Data points show individual donors. **D)** Collagen uptake by flow cytometry of LPS-stimulated macrophages treated with the dynamin inhibitor Dynasore (n = 3, One-way ANOVA with a Dunnett's multiple comparisons test). Data points show individual donors. **E)***x*-*y* projected area from confocal microscopy images of intracellular storage compartments containing FITC collagen (see Supplementary Fig. 1B). Every data point represents one cell (pooled from *n* = *4* donors, unpaired *t*-test). (For interpretation of the references to colour in this figure legend, the reader is referred to the Web version of this article.)

We developed an assay for the quantitative determination of fibrous collagen uptake by hMDMs. We coated wells with FITC-labelled collagen type I, the most abundant ECM component in the human body [22] (Fig. 1A). Confocal *z*-stacks of live macrophages treated for 24 h with LPS (100 ng/ml) and cultured on these collagen surfaces showed that the collagen was ingested, because it was present in intracellular compartments (Fig. 1B, yellow arrows). To quantify this collagen uptake, we detached the hMDMs from the collagen surface after LPS-treatment, washed away the extracellular collagen, and measured the levels of ingested FITC-collagen by flow cytometry (Fig. 1C; gating in Supplementary Fig. 1A). Similar results were obtained with accutase and trypsin, showing that the quantification of collagen uptake was not affected by the method of detachment. To verify that the FITC-collagen levels were not due to extracellular collagen adhering to the cell surface, we also performed control experiments with Dynasore, a small molecule inhibitor of the dynamin GTPase which is responsible for membrane scission during endocytosis (Fig. 1D). These results demonstrate that LPS promotes the uptake of fibrous collagen by endocytosis in hMDMs.

Macrophages can degrade ECM components like collagen by uptake and proteolytic processing [23,24]. Immunostaining for the key endo/lysosomal protease cathepsin B showed that the ingested collagen reached degradative compartments (Supplementary Fig. 1B). The control macrophages showed a lower staining intensity of cathepsin B, likely because inflammatory activation increases its expression [25]. Quantification of the size of these collagen-containing compartments (i.e., surface area projected in the x/y-plane of the microscopy images) also indicated that LPS-stimulated hMDMs had more intracellular collagen (Fig. 1E), strengthening our conclusion from that LPS promotes the uptake of fibrous collagen. Thus, LPS activated hMDMs have increased collagen uptake in proteolytic lysosomal compartments.

### 2.2 IRG1 reduces TNF-α secretion and collagen uptake in pro-inflammatory macrophages

**Fig. 2:**
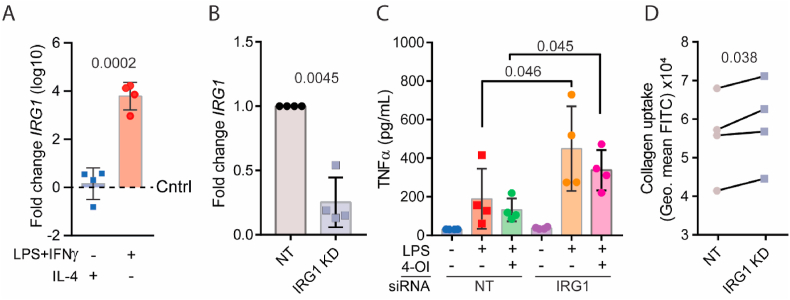
***IRG1* knockdown promotes TNF-α secretion and increases collagen uptake by human blood monocyte derived macrophages (hMDM). A)** mRNA levels (log10) of *IRG1* in pro- (LPS and IFNγ) and anti-inflammatory (IL-4) macrophages after 24 h of polarisation normalised to untreated macrophages (Cntrl; dashed line). **B)** qPCR of *IRG1* mRNA levels after IRG1 knockdown and 24 h of LPS stimulation, normalised to the non-targeted siRNA control (NT) (*n* = *4*, paired *t*-test). **C)** ELISA of secreted TNF-α at 24 h after stimulation of macrophages with LPS, 4-OI or both, and with or without IRG1 knockdown (*n* = *4*, paired *t*-test). **D)** Collagen uptake by flow cytometry of macrophages that were incubated for 24 h in FITC-collagen-coated wells with LPS after IRG1 knockdown (*n* = 4 donors, paired *t*-test).

Compared to alternatively activated hMDMs, which are differentiated with interleukin (IL)-4 and have an anti-inflammatory phenotype, conventionally activated hMDMs, which are polarised in the presence of LPS and the inflammatory cytokine interferon (IFN)-γ, have a ∼10,000-fold elevated expression of *IRG1*, the gene responsible for itaconate production (Fig. 2A). To evaluate the role of *IRG1* in inflammatory macrophages, we performed a knockdown of *IRG1* with siRNA (Fig. 2B), stimulated the cells with LPS, and measured TNF-α secretion. *IRG1* knockdown resulted in elevated LPS-induced production of TNF-α (Fig. 2C). To investigate whether itaconate could rescue the knockdown of IRG1, we also added the membrane-permeable itaconate derivative 4-OI (100 μM), which showed a slight reduction in TNF-α production (albeit not significant). In addition, we found that *IRG1* depleted macrophages ingested larger amounts of collagen (Fig. 2D). This prompted us to further investigate the role of 4-OI in collagen uptake by hMDMs.

### 2.3 4-OI inhibits inflammation via increased NRF2 and reduced p38 signalling

**Fig. 3:**
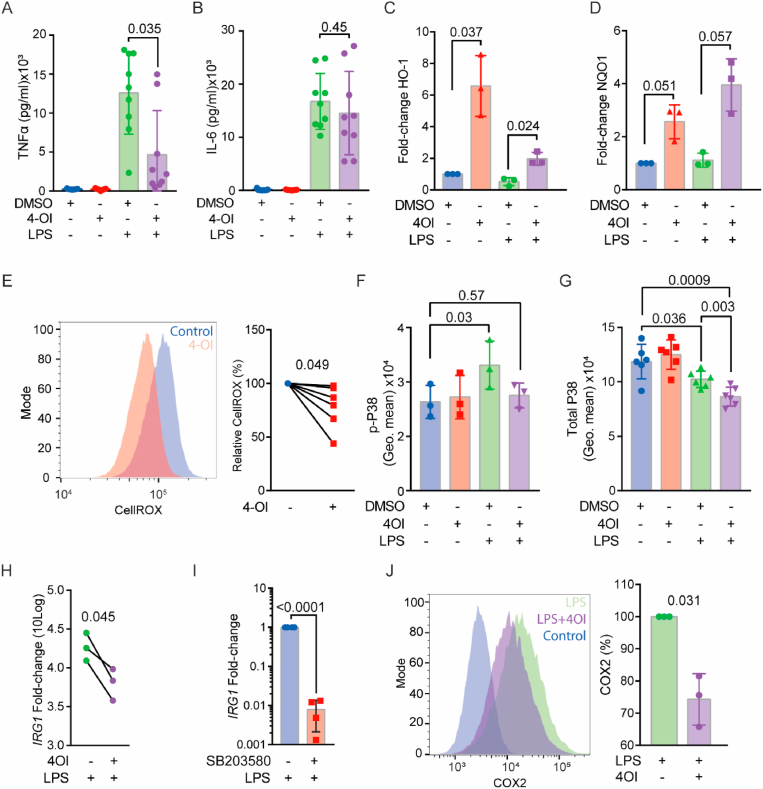
**4-OI increases NRF2 and decreases p38 MAPK signalling in human blood monocyte derived macrophages (hMDM) and downregulates pro-inflammatory markers. A-B)** ELISA of secreted TNF-α **(A)** or IL-6 **(B)** at 24 h after stimulation of LPS, 4-OI or both (*n* = 9 donors, paired *t*-test). Data points show individual donors. **C)** mRNA levels of downstream NRF2 targets heme oxygenase-1 (*HO1*) and **D)** NAD(P)H quinone dehydrogenase (quinone 1) (*NQO1*) after stimulation of macrophages for 6 h with either LPS, 4-OI or both. DMSO: solvent control (*n* = 3 donors, one-way ANOVA with a Tukey's multiple comparison test). **E)** Representative flow cytometry histogram and quantification of cellular ROS levels by staining with CellROX after 24 h of 4-OI treatment (*n* = 6 donors, paired *t*-test). **F)** Phospho-flow of phosphorylated-p38 MAPK (Thr180/Tyr182) immunostaining of macrophages at 1 h after stimulation with LPS, 4-OI or both (*n* = 3 donors, one-way ANOVA with a Dunnette's multiple comparison test). **G)** Total intracellular p38 staining at 1 h after stimulation with LPS, 4-OI or both (*n* = 6 donors, one-way ANOVA with a Tukeys multiple comparison test).**H)***IRG1* levels of macrophages stimulated with LPS, with and without 4-OI, after 24 h normalised to controls without LPS (*n* = 3 donors, paired *t*-test). **I)** mRNA levels of *IRG1* after LPS stimulation for 24 h with and without the p38 inhibitor SB203580 (*n* = 4 donors, paired *t*-test). **J)** Representative flow cytometry histogram and quantification of internal COX-2 levels at 24 h of LPS and 4-OI stimulation (*n* = 3 donors, paired *t*-test).

First, we determined the effect of 4-OI on the inflammatory state of LPS-stimulated macrophages. We measured the production of TNF-α and interleukin-6 (IL-6) in LPS-stimulated hMDMs (Fig. 3A). TNF-α production was inhibited by 4-OI (100 μM), in accordance with the results of the IRG1 knockdown experiments. However, we did not observe a reduction of IL-6 secretion by hMDMs (Fig. 3B). This treatment also did not influence the viability of the cells (Supplementary Fig. 2A).

4-OI has been shown to induce NRF2 signalling in human macrophages [26]. To confirm this, we treated hMDMs for 6 h with 4-OI and/or LPS and measured the expression of downstream genes of NRF2 signalling heme oxygenase-1 (*HO1*) and NAD(P)H quinone dehydrogenase 1 (*NQO1*) by qPCR (Fig. 3C–D). 4-OI increased mRNA levels of both *HO1* and *NQO1*, thus confirming that 4-OI promotes the NRF2 pathway. The NRF2 pathway is involved in the cellular defence against oxidative stress by decreasing reactive oxygen species (ROS). To determine whether 4-OI lowered the levels of ROS in the hMDMs, we conducted experiments with the fluorescent ROS probe CellROX (Fig. 3E). As reported previously [27], we found that 4-OI reduced the total levels of ROS in hMDMs.

As ROS is an important signalling molecule in the activation of the p38 pathway, a mitogen-activated protein kinase (MAPK) signalling pathway controlling inflammatory cytokine production in macrophages [28,29], we considered the possibility that 4-OI could decrease p38 signalling. To determine this in human macrophages, we stimulated the hMDMs for 1 h with LPS and measured levels of phosphorylated p38 (p-p38; Thr180/Tyr182) using phospho-flow (Fig. 3F; image of staining Supplementary Fig. 2B). Indeed, we found that 4-OI completely reverted the LPS induced increase in p-p38 levels. We also stained for the total levels of p38 (Fig. 3G), demonstrating that LPS stimulated hMDM have decreased levels of p38 after 1 h of treatment, but even more reduced when 4OI was combined with LPS.

To further confirm that 4-OI inhibits p38 signalling, we also measured *IRG1* mRNA levels (Fig. 3H; same samples as Fig. 3C–D), because the stability of this mRNA depends on p38 signalling [30]. Strengthening our conclusion that the decreased of *IRG1* levels by 4OI was caused by lower p38 activity, we found that the p38 inhibitor SB203580 also lowered *IRG1* mRNA levels in hMDMs (Fig. 3I). We also measured protein levels of cyclooxygenase-2 (COX-2), an enzyme downstream of p38 signalling involved in the production of prostaglandins [29]. As expected [26], 4-OI decreased COX-2 levels in hMDMs, as determined by flow cytometry (Fig. 3J).

In murine dendritic cells, IRG1 has been shown to regulate communication with T cells by decreasing expression of the T cell-activating factor CD80 [31]. To investigate the effect of 4-OI on the expression of T cell activation factors in hMDMs, we measured surface levels of CD80 and major histocompatibility complex (MHC) class II by flow cytometry (Supplementary Figs. 2C–D). However, we did not observe alteration of these surface markers by 4-OI. Thus, 4-OI does not inhibit all inflammatory markers of macrophages, as we only found a reduction of TNF-α and COX-2, but not of IL-6, CD80 and MHC class II. Moreover, 4-OI inhibits p38 activity and upregulates NRF2 signalling.

### 2.4 4-OI promotes a pro-resolution macrophage phenotype in hMDMs

**Fig. 4:**
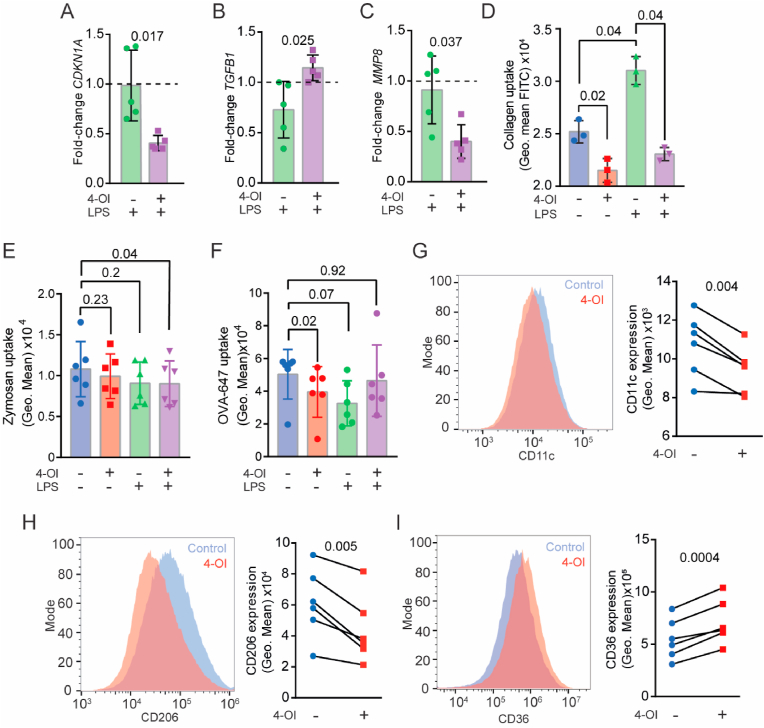
**4-OI upregulates pro-resolution macrophage markers and reduces collagen uptake by human blood monocyte derived macrophages (hMDM). A-C)** qPCR of cyclin dependent kinase inhibitor 1A (*CDKN1A*) **(A)**, transforming growth factor beta-1 (*TGFB1*) **(B)** and MMP8 **(C)** mRNA levels after 24 h of LPS stimulation with and without 4-OI, normalised to an untreated control (dashed line) (*n* = *5*, paired *t*-test). **D)** Collagen uptake by flow cytometry of macrophages that were incubated for 24 h in FITC-collagen-coated wells with 4-OI, LPS or both (*n* = 3 donors, one-way ANOVA with a Dunnette's multiple comparison test). **E)** FITC-labelled zymosan uptake by macrophages after 24 h of incubation with LPS, 4-OI or both (*n* = 6, one-way ANOVA with a Dunnett's test for multiple comparison). **F)** Alexa Fluor 647-labelled ovalbumin (OVA-647) uptake by macrophages after 24 h of incubation with LPS, 4-OI or both (*n* = 6, one-way ANOVA with a Dunnett's test for multiple comparison). **G-H)** Representative flow cytometry histograms and quantifications of surface levels of collagen receptors and M2-macrophage markers CD11c (**G**), CD206 (**H**), and CD36 (**I**) in hMDMs treated with 4-OI for 24 h (*n* = 6 donors, paired t-tests). Data points show individual donors.

Because our data suggest that 4-OI reduces the pro-inflammatory phenotype of the hMDMs, and we observed that IRG1 knockdown resulted in higher collagen uptake, we hypothesised that 4-OI might drive the pro-inflammatory hMDMs to a pro-resolution phenotype. Inflammation can increase cellular senescence, as this is induced by an increase of ROS production [32,33]. Cellular senescence can impair the macrophage functions in wound healing and tissue homeostasis [34]. Therefore, we first investigated the effect of 4-OI on the mRNA levels of the senescent marker P21 (*CDKN1A*) [35] (Fig. 4A). We found that 4-OI treatment reduces the expression of *CDKN1A* by about 50%. TGF-β plays key roles in wound healing, as it stimulates angiogenesis, fibroblast proliferation, collagen synthesis and the deposition and remodelling of ECM [36]. For instance, loss of TGF-β has been reported in chronic non-healing wounds [37,38]. Therefore, we also investigated the effect of 4-OI on expression of *TGFB1* by qPCR (Fig. 4B). We found that the mRNA levels of *TGFB1* were increased in 4-OI and LPS treated hMDMs. However, we did not observe a significant increase upon knockdown of *IRG1* (Supplementary Fig. 3A), possibly due to the dual role of itaconate in inflammation and/or insufficient knockdown efficiency. During inflammation, the surrounding tissue is degraded by matrix metalloprotease (MMP). In particular, MMP8 is a collagenase found in tissue of wounds that have reduces healing [39]. Because MMP8 is also secreted by macrophages [40], we determined transcript levels of *MMP8* by qPCR (Fig. 4C). These results show that 4-OI reduces *MMP8* mRNA levels by about 50%. However, it should be noted that human blood monocytes also secrete other MMPs like MMP-2 and -9[41,42] in proinflammatory conditions, which might also affect collagen uptake. Moreover, macrophage also produce vascular endothelial growth factor (*VEGF*) which promotes wound healing by increasing angiogenic blood vessel formation [43], but we did not observe any effect of 4-OI on the expression of *VEGF-A* (Supplementary Fig. 3B). However, it should be noted that these findings are all at the mRNA level and do not necessarily correspond with protein levels.

Next, we investigated whether 4-OI would affect the uptake of fibrous collagen by hMDMs (Fig. 4D). Our results show that 4-OI reduced collagen uptake by the hMDMs and completely blocked the LPS-mediated increase of collagen uptake, in accordance with our results with the IRG1 knockdown. To determine whether this inhibition of collagen uptake by 4-OI was specific or whether this was a broad endocytic effect, we also measured uptake of fluorescently-labelled zymosan (i.e., killed yeast particles) and the protein ovalbumin (Fig. 4E–F). 4-OI did not inhibit uptake of these compounds in LPS stimulated cells, indicating that 4-OI specifically reduces collagen uptake. However, in absence of LPS, 4-OI reduced uptake of ovalbumin. To investigate if receptors involved in collagen uptake like the integrin CD11c and the mannose receptor CD206 are affected by 4-OI, we measured the surface levels of these receptors on the hMDMs (Fig. 4G–H). We detected a decrease in the levels of both CD11c and CD206 by 4-OI. As the mannose receptor CD206 also mediates OVA uptake, this might explain the reduced uptake of ovalbumin [44].

Because CD206 and TGF-β are canonical markers for M2 macrophages, and 4-OI was recently shown to down-regulate M2 polarisation by decreasing JAK1 and STAT6 signalling [45], we determined the effects of 4-OI on the other M2 marker CD36. CD36 is reported to be upregulated in macrophages during the later phases of wound healing (day 14) [17] and is found to play a role in tissue healing by acting as a scavenger receptor [46]. In accordance with our findings of TGF-β, we found that 4-OI increases CD36 compared to untreated hMDMs (Fig. 4I). These results support our conclusion that itaconate inhibits collagen uptake and steers the hMDM to a pro-resolution M2-like phenotype hallmarked by the upregulation of TGF-β and CD36 and the downregulation of CD11c and CD206.

### 2.5 4-OI inhibits glycolysis in combination with LPS which is important for collagen uptake

**Fig. 5:**
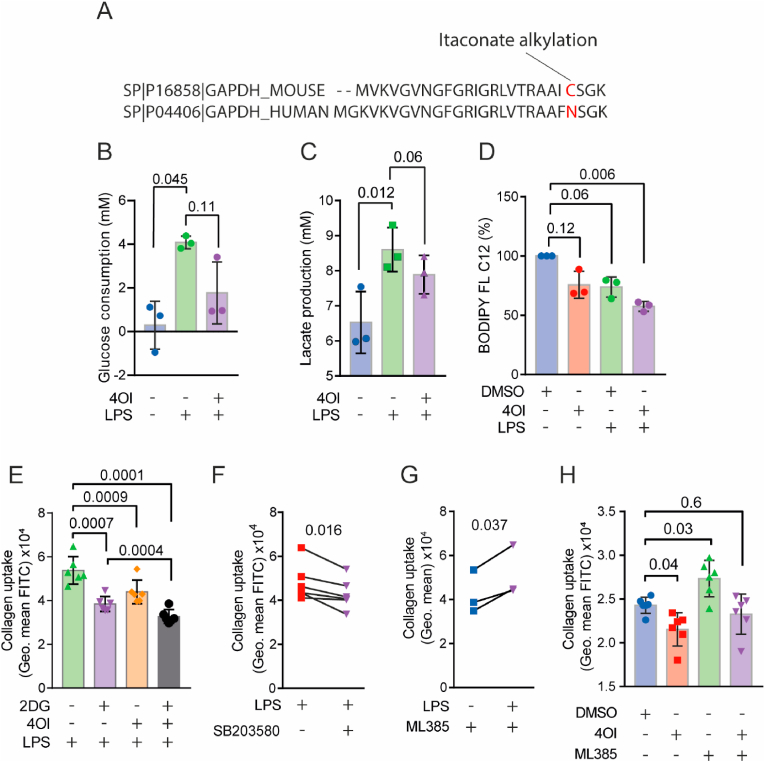
**4-OI blocks collagen uptake by promoting NRF2 and inhibiting p38 signalling in human blood monocyte derived macrophages (hMDM). A)** Sequence alignment of mouse and human GAPDH. Red: murine cysteine 22 alkylation site. **B)** Glucose reduction of media in which hMDMs were cultured and treated with LPS or LPS and 4-OI (*n* = 3 donors, paired *t*-test). **C)** Lactate production in media in which hMDMs were cultured and treated with LPS or LPS and 4-OI (*n* = 3 donors, one-way ANOVA with a Tukey's multiple comparison test). **D)** Percentage BODIPY FL C_12_ uptake 24 h after incubation with LPS, 4-OI or both with flow cytometry (*n* = 3 donors, one-way ANOVA with a Dunnette's multiple comparison test). **E)** Quantification of fibrous FITC-collagen uptake by hMDMs treated with glycolysis inhibitor 2-deoxy glucose (2DG), LPS and/or 4-OI for 24 h by flow cytometry. (*n* = 6 donors, one-way ANOVA with a Tukey's multiple comparison test). **F)** Uptake of fibrous FITC-collagen by LPS-stimulated hMDMs (24 h) incubated with p38 MAPK inhibitor SB203580 by geometric mean with flow cytometry (*n* = 6 donors, paired *t*-test). **G)** Collagen uptake by LPS-stimulated hMDMs (24 h) with NRF2 inhibitor ML385 (*n* = 3 donors, paired *t*-test). **H)** Quantification of fibrous FITC-collagen uptake by macrophages treated with ML385 and/or 4-OI by flow for 24 h cytometry. DMSO: solvent control. (*n* = 6 donors, one-way ANOVA with a Dunnette's multiple comparison test). Data points show individual donors. (For interpretation of the references to colour in this figure legend, the reader is referred to the Web version of this article.)

We recently showed that inhibition of glucose metabolism can influence collagen uptake by adding 2-deoxy glucose (2-DG) to hMDMs [47], which we repeated in this study (Supplementary Fig. 4A). Since itaconate has been shown to reduce glucose metabolism of murine macrophages [48], we decided to investigate the role of 4-OI on the inflammation-induced glycolytic flux in hMDMs. This flux in inflammation is generally associated with increased inflammatory signalling and immune effector functions in macrophages. Itaconate is known to alkylate the cysteine-22 residue of murine GAPDH [21,27], however, this cannot occur in humans because the cysteine residue is not conserved (Fig. 5A). We therefore quantified the uptake of the fluorescent glucose analogue 2-(N-(7-nitrobenz-2-oxa-1,3-diazol-4-yl)amino)-2-deoxyglucose (2-NBDG). 2-NBDG uptake was two-fold increased by 4-OI during LPS stimulation (Supplementary Fig. 4B). However, we did not observe an increase on 2-NBDG uptake by LPS stimulation of the hMDMs, similar to previous studies in hMDMs [49,50]. 2-NBDG uptake is not a reliable tool to assess glucose uptake and additional experiments are required to confirm this result [51]. Therefore, we performed post-incubation glucose and lactate measurements on supernatants. Here, we observed that LPS-stimulated hMDMs consumed more glucose and 4-OI non-significantly inhibited this (Fig. 5B). However, hMDMs with *IRG1* siRNA knockdown (i.e., with lower itaconate production) showed significantly lower glucose consumption (Supplementary Fig. 4C), opposite to our expectations based on 4-OI.

Pro-inflammatory macrophages also have increased aerobic glycolysis to meet the metabolic requirements of inflammation. Therefore, we also measured lactate production of hMDMs treated with LPS and 4-OI (Fig. 5C). Similar to glucose consumption, lactate production was increased upon LPS stimulation and again was non-significantly reduced by 4-OI. However, lactate production of hMDMs with siRNA knockdown for *IRG1* was not affected (Supplementary Fig. 4D). These results are contradicting regarding the regulation of glycolysis by itaconate. We speculate that, since the multifaced itaconate also promotes the activation of macrophages with pro-inflammatory stimuli by activation [52,53], rearranges the TCA cycle [53], and simultaneously reduces the glucose metabolism pathways [48], this mechanism is likely more complex than just the inhibition of GAPDH. We also tested fatty acid uptake with the BODIPY FL C_12_ probe. We found reduced uptake by about 40% when hMDM were cultured with both 4OI and LPS-stimulated hMDMs compared to the control condition (Fig. 5D).

As mentioned above, the glycolytic flux supports the pro-inflammatory function of macrophages. Moreover, glucose also plays a role in trafficking of receptors like CD206 [54]. To further assess if the reduced glucose metabolism contributes to the 4-OI-mediated reduction of collagen uptake, we treated hMDMs with 2-DG during LPS stimulation (Fig. 5E). In line with our previous observation, we found that LPS-stimulated macrophages treated with 2-DG have reduce collagen uptake. However, we observed an additional inhibition of collagen uptake by the combination of 4-OI and 2-DG compared to these compounds alone (Fig. 5E). These cumulative effects of 4-OI and 2-DG suggest that 4-OI additionally inhibits collagen uptake via a mechanism independently of glycolysis.

### 2.6 4-OI inhibits collagen uptake via NRF2 and p38 signalling

In the final set of experiments, we investigated whether the downregulation of collagen uptake by 4-OI was attributable to its effects on p38 and/or NRF2 signalling. We studied these effects using the small molecule inhibitors ML385 (NRF2) and SB203580 (p38) and our flow cytometry-based assay for collagen uptake. The p38 inhibitor reduced collagen uptake in combination with LPS, but not in the absence of LPS (Fig. 5F; Supplementary Fig. 4E). In contrast, the NRF2 inhibitor ML385 increased collagen uptake by about 21% during LPS stimulation (Fig. 5G). In fact, even in the absence of LPS, the NRF2 inhibitor increased collagen uptake and completely blocked the inhibition of collagen uptake by 4-OI (Fig. 5H). ML385 also decreased expression of the gene *HO-1* (Supplementary Fig. 4F), confirming the successful inhibition of NRF2. Moreover, neither 4OI nor ML385 affected uptake of latex beads, strengthening our conclusion that the effects on collagen uptake are not a general phagocytic effect (Supplementary Fig. 4G). These findings indicate that itaconate inhibits collagen uptake by reducing p38 activity and increasing NRF2 signalling.

## 3 Discussion

Macrophages are important immune cells in the pathology of wounds as they are involved in almost all stages of wound healing: They play key roles in inflammation, reparation of tissue, resolution of inflammation, removal of apoptotic bodies, angiogenesis, and promotion of ECM synthesis and remodelling [3]. To enable these versatile and often contrasting functions, macrophages need to be extremely plastic and adapt their phenotype during different stages of wound healing. Knowledge of the factors that shape the macrophage phenotype is essential for the development of new therapies for diseases and disorders associated with dysfunctional wound healing and fibrosis. We found that inflammatory activation promotes the uptake of collagen type I by macrophages. This likely contributes to the impaired wound healing in pathologies like chronic wounds. The concept is emerging that the accumulation of itaconate following the upregulation of *IRG1* is important for the pro-inflammatory functions of macrophages. For instance, itaconate is responsible for the inhibition of complex II (succinate dehydrogenase) resulting in increased succinate production and this supports inflammatory cytokine production [16]. Moreover, in myeloid cells, itaconate inhibits growth of the intracellular pathogen *Salmonella* [55], whereas extracellular itaconate can also impair bacterial growth by targeting their metabolism [56,57]. However, itaconate can also dampen inflammation as it targets KEAP1, thereby upregulating the NRF2 pathway [27]. In this study, we show that the late-phase production of itaconate contributes to a pro-resolution phenotype in hMDMs, because it inhibits collagen uptake and promotes expression of *TGFB1* and reduces *MMP8*.

We found that 4-OI, a membrane permeable derivative of itaconate, inhibits collagen uptake by promoting the NRF2 pathway and reducing p38 signalling, because we found that (i) 4-OI promotes NRF2 signalling, (ii) 4-OI inhibits p38 signalling, and (iii) collagen uptake could be blocked by inhibition of NRF2 and p38. An open question is how NRF2 and p38 signalling affect collagen uptake. This effect is not attributable to an overall reduction of endocytosis or cytoskeletal remodelling, since the uptake of zymosan and latex beads was not affected by 4-OI. Therefore, it seems more likely that 4-OI directly affects collagen uptake by its receptors. Indeed, we found that 4-OI reduced the surface expression of the known collagen receptors CD11c and CD206. It might be that 4-OI also affects other collagen receptors, like the discoidin domain receptor 1[58].

IRG1 is upregulated during the early stages of wound-healing (day 4) in wound-resident macrophages in mouse [14]. Since we found that itaconate (*i.e.,* the product of IRG1) reduces collagen uptake in hMDMs, this suggests that itaconate could diminish the uptake of collagen by infiltrating macrophages to promote healing. During later stages of wound healing, more anti-inflammatory macrophages (also known as alternatively activated macrophages) are present in wounds. 4-OI and itaconate have been shown in mouse to inhibit the polarisation of macrophages to an anti-inflammatory (IL-4 and IL-13) phenotype, by the inhibition of JAK1 and STAT6 [45]. These macrophages also decreased their surface levels of CD206, as we observed in macrophages treated with 4-OI without IL-4 or IL-13 (which was independent of NRF2). This suggests that itaconate might also regulate collagen remodelling during the latter stages of wound healing. Furthermore, we found that inflammatory macrophages can ingest collagen type 1; to our knowledge this finding had not been shown anywhere to date. Since chronic wounds have substantial macrophage infiltration, this might potentially contribute to the impaired healing of chronic wounds.

FYVE, RhoGEF and PH domain-containing protein 6 (FDG6) might also be involved in the specific regulation of collagen uptake. FDG6 is alkylated on Cys954 by 4-OI in mouse (conserved Cys984 in humans) according to mass spectrometry data [27]. Moreover, FDG6 is a Ras-like family of Rho- and Rac proteins and involved in actin mediated processes like macropinocytosis via CDC42 [59] and endosome recycling of receptors. If itaconate inhibits the function of FDG6 by modification of Cys984, this might also contribute to the reduced uptake of collagen.

Glycolysis regulation by itaconate has been well documented in mouse macrophage literature [21,60,61]. In brief, itaconate alkylates the proteins in the glycolytic pathway like GAPDH, or fructose-bisphosphate aldolase A in murine macrophages. This makes the macrophage less oriented towards glycolysis. Our results in hMDMs with 4-OI partially support these findings, even though the Cys22 in human GAPDH is not conserved. Although we found a trend in reduced glucose uptake and lactate production when treated with 4-OI; the knockdown of *IRG1* did not increase glycolysis. This indicates that the metabolic regulation of glucose uptake by macrophages is perhaps regulated on more levels than just GAPDH. For instance, itaconate also alkylates fructose-bisphosphate aldolase A^48^. Furthermore, itaconate has also shown to regulate the glycolytic state of macrophage via MyD88 [60]. Moreover, it should also be noted that 4-OI and itaconate have some discrepancies in their alkylation profile [7]. Finally, the fact that itaconate also contributes to promoting early inflammation by inhibition of succinate dehydrogenase [53] might also be a consequence of this contradicting result.

In addition to its effects on NRF2 and p38 signalling and inhibition of collagen uptake, 4-OI also has other effects. We found that it lowers ROS levels, reduces TNF-α production, reduces COX-2 and p21 expression, shifts the macrophage metabolism from lipid to sugar uptake, and increases the expression of the cytokine TGF-β. These effects are generally considered anti-inflammatory and promote wound healing. In fact, NRF2 [62] and p38 [63] are already considered therapeutic targets in wound healing. For instance, NRF2 is part of an integrated signalling pathway involved in wound healing in mouse macrophages, together with NF-κB and SMAD3 (down-stream of TGF-β) [64]. Likewise, p38 signalling has many effects in the inflammatory NF-κB pathway and ECM-processing, as it for example promotes expression of TNF-α and metalloproteases [65]. However, p38 also promotes the expression of *IRG1*, suggesting a feedback loop where the p38 signalling is dampened by itaconate production. This suggests that a strong initial p38 activation is required for the progression into a wound healing phenotype by increasing late stage IRG1 expression and itaconate production. 4-OI can thus be expected to promote the wound healing process by inhibiting p38 and promoting NRF2 signalling. Thus, our study shows an important role for *IRG1* and itaconate in the uptake of collagen and suggests that 4-OI might be a candidate drug for treatment of pathological conditions associated with wound healing, perhaps, also given its anti-microbial properties [9], as topical medication.

## 4 Materials and methods

### 4.1 Materials

4-octyl itaconate (Sigma, #sml2338), SB203580 and ML385 (MedChemExpress) were all dissolved in anhydrous DMSO (Sigma, #276855). LPS (*E.coli* OB111, Sigma #L4391) was dissolved in PBS (Dulbecco).

### 4.2 Culture of hMDMs

Approval to conduct experiments with human blood samples was obtained from the Netherlands blood bank Sanquin and all experiments were conducted according to national and institutional guidelines. Informed consent was obtained from all blood donors by Sanquin. Samples were anonymized and none of the investigators could discover the identity of the blood donors.

Monocytes were isolated from human buffy coats using CD14^+^ MACS (Miltenyi). 5 × 10^6^ cells were cultured in Ultra-low adherent 6-well plates (Corning) in RPMI containing 10% FBS (Hyclone), glutamine (Lonza), antibiotics (Gibco), and human macrophage-colony stimulating factor (M-CSF)(R&D 216-MC, 100 ng/ml) at 37 °C and 5% CO_2_ for seven days. On day 4, macrophages were supplemented with medium containing 50 ng/ml M-CSF. After differentiation, the cells were washed once with room temperature PBS (Gibco) followed by the addition of cold (4 °C) PBS and incubated at 4 °C for 30 min to collect the macrophages.

For stimulation of macrophages, LPS (OB111 *E. coli*; Sigma) was used at 100 ng/ml for 6 or 24 h 4-OI (100 μM) was added separately prior to the prompt addition of LPS.

### 4.3 Knockdown of *IRG1*

Knockdown of IRG1 was conducted with the Neon transfection system (Fisher Scientific, 10090314) using the 10 μl tip protocol for siRNA (1000 V, 40 ms, 2 Pulses). Using 20 picomol per transfection, 2–3 x 10^6^ cells were transfected either with IRG1 ON-TARGETplus siRNA (Horizon Discovery Biosciences Ltd, L-180668-01-0005) or siRNA negative control (Thermo Scientific, 12935300/10143902) in T-buffer and transferred to an ultra-low adherence plate (Corning, 3471) into RPMI without phenol red (Gibco, ThermoFisher, 11835030), containing glutamine (Thermo Scientific, 15430614), 10% FBS (Hyclone, ThermoFisher, 10309433) and 50 ng/ml M-CSF for overnight recovery at 37 °C and 5% CO_2_. The next day, samples were removed from the plate and used for collagen uptake, ELISA and qPCR as described below.

### 4.4 Confocal microscopy

For live-cell imaging, cells were seeded (100,000 cells per quadrant) on Cellview culture dishes (Greiner bio-one, 627870) which were coated with 10 μg/ml FITC-labelled collagen (Sigma, C4361) in PBS for 1 h at room temperature in the dark. Cells were stimulated with LPS (100 ng/ml) for 24 h.

After collagen uptake in a 24 well plate (coated as described above) macrophages were seeded on glass coverslips in RPMI for 30 min to attach and fixed using 4% paraformaldehyde (PFA, 15 min at 4 °C) followed by four PBS washes. Cells were blocked and permeabilised for 30 min at 4 °C with CLSM buffer (PBS + 20 mM glycine + 3% BSA) and 0.1% saponin followed by an overnight staining with the primary antibody (Cathepsin B, Calbiochem IM27L and LAMP1, Biolegend 328601) 1:200 diluted in CLSM with saponin at 4 °C. The next day cells were washed twice with PBS +0.1% saponin and incubated for 30 min at room temperature in CLSM +0.1% saponin with secondary antibodies donkey-anti-mouse IgG (H&L) labelled with Alexa 647 and donkey-anti-rabbit with Alexa 568. After washing the cells with PBS with 0.1% saponin, the coverslips were mounted on glass slides in 67% glycerol containing Trolox (1 mM) and DAPI (0.33 μg/ml). Samples were imaged using an LSM800 Zeiss microscope with a 63 × oil immersion lens.

### 4.5 Quantitative PCR

RNA was isolated with the Quick-RNA miniprep kit (R1055A) according to company protocol. After RNA-isolation cDNA synthesis was conducted with random hexamer primers (Roche, 11034731001) with the M-MLV Reverse Transcriptase kit (ThermoFisher, 28025013). cDNA was diluted in Ultra-pure water (Gibco) to 0.66 ng/μl for RT-qPCR with Power up SYBR Green Mastermix and the 10 μl/reaction protocol (Thermo Scientific, A25741) using the following primers for each target gene: *SNRPD3* (ref gene) (FWD: 5’ GGAAGCTCATTGAAGCAGAGGAC 3’ RV: 5’ CAGAAAGCGGATTTTGCTGCCAC 3’), *IRG1* (FWD: 5’ TGTGAACGGTGTGGCTATTCA 3’ RV: 5’ AGGGGGATGGAATCTCTTTGG 3’) *HO1* (FWD: 5’ CCAGGCAGAGAATGCTGAGTTC 3’ RV: 5’ AAGACTGGGCTCTCCTTGTTGC 3’), *NQO1* (FWD: 5’ CCTGCCATTCTGAAAGGCTGGT 3’ RV: 5’ GTGGTGATGGAAAGCACTGCCT 3’), *TGFB1* (FWD 5’ GCAAGTGGACATCAACGGG 3’ RV: 5’ TCCGTGGAGCTGAAGCAATA 3’), *VEGF-A* (FWD 5’ CATGCAGATTATGCGGATCAA 3’ RV: 5’ TTTGTTGTGCTGTAGGAAGCTCAT 3’) with a BioRad CFX96 qPCR System.

### 4.6 Flow cytometry

For live-cell flow cytometry, differentiated macrophages were seeded at 10^5^ cell/well in a 96-well ultra-low adherence plate (Corning) and treated with LPS, 4-OI or both in a CO_2_ incubator (5%) at 37 °C for 24 h. After treatment, cells were incubated with CellROX (5 μM), zymosan-FITC (used at 5 particles per cell) or ovalbumin-Alexa Fluor 647 (10 μg/ml, ThermoFisher, O34784), Latex FluoSpheres 565/580 (Molecular probes, F-8802) for 30 min, or 2-NBDG (50 μM, Thermo Scientific, N13195/11569116) or BODIPY FL C12 (1 μM, Thermo Scientific, D3822/10615583) for 15 min, collected and resuspended in a V-bottom 96-well plate. Live-cell flow cytometry (CytoFlex S, Beckman Coulter) was conducted in warm RPMI without phenol red.

For cell surface receptors, cells were transferred to a 96 well V-bottom plate after treatment and blocked with 2% human serum in PBS for 30 min at 4 °C. Next CD11c-APC (BD, 559877), HLA-DR-APC (BD, 559866), CD80-APC (BioLegend, 305220), or CD206-APC (BioLegend, 321110) antibodies were added (0.5 μl/10^5^ cells) for 30 min in 2% human serum in PBS. After staining, cells were washed twice with PBS and analysed on a CytoFLEX S from Beckmann coulter.

For internal staining, after treatment, cells were transferred to a 96 well V-bottom plate, stained with e780 fixable live/dead staining (1:1,000, 15 min at 4 °C), fixed in 4% PFA, and washed 4 times with PBS. Next, cells were permeabilised and blocked with 2% human serum and 0.05% saponin in PBS for 30–60 min at 4 °C. Antibody (Clone AS67, BD Biosciences #565125) staining for COX-2 was done in PBS with 2% human serum and 0.05% saponin using 0.5 μl antibody per well for 30–60 min at 4 °C. After staining, cells were washed twice with PBS and analysed on the CytoFLEX S.

For Phospho-flow, samples were stained with e780 and fixed with 4% PFA and washed 4 times with tris-buffered saline (TBS, pH 7.5). Next, cells were permeabilised and blocked with 2% human serum and 0.5% Triton-X in TBS for 60 min. Samples were stained with p-p38 antibody (1:400, cell signalling #9211S) in TBS with 2% human serum for 30 min, in the dark, at room temperature. Cells were then washed twice with TBS containing 2% human serum and stained with a secondary donkey-anti-mouse IgG (H&L) labelled with Alexa fluor 488, for another 30 min. After this, the cells were washed once more in TBS and analysed on a CytoFLEX S. In parallel, cells with similar treatment on glass cover slips were stained exactly the same, to verify nuclear staining of the p-p38 antibody.

### 4.7 Collagen uptake assay

A 24-wells plate was coated with 10 μg/ml FITC-labelled collagen (Sigma, C4361) in PBS for 1 h at room temperature in the dark. After incubation, wells were twice washed with PBS. Next, 100,000 macrophages were seeded into the wells and cultured for 24 h with LPS, 4-OI, or both (adding 4-OI 1 min prior to LPS). The next day, the samples were washed with PBS and macrophages were detached using StemPro Accutase (Fisher Scientific, 11599686) for 10 min in the cell culture incubator followed by a e780 fixable live/dead staining (1:1,000, 15 min at 4 °C), fixation in 4% PFA, and analysis by flow cytometry. The addition of inhibitors SB203580 (10 μM) and ML385 (10 μM) in DMSO was done prior to the addition of cells and supplementation with 4-OI or LPS.

### 4.8 ELISA

Macrophages were seeded at 30,000 cells per well in a flat-bottom 96-well plate and stimulated with 4-OI (100 μM) and LPS (100 ng/ml). Media was taken from samples after 24 h and stored at −20 °C. TNF-α and IL-6 ELISA was performed according to company protocols (Fisher Scientific, 15561127).

### 4.9 Glucose consumption assay

Total glucose content was measured from supernatant of 4-OI (100 μM) and LPS (100 ng/ml) treated, as well as *IRG1* siRNA transfected macrophages. 250 μL of glucose reagent from the BIOLABO GOD-PAP glucose kit was pipetted into a 96-well plate. Samples and standard (5 μl per well) we added to the reagent, mixed by pipetting and incubated for 30 min in the dark. Absorbance was measured at 490 nm. Glucose consumption was calculated as the difference between glucose levels of medium without cells and glucose levels in cell supernatants.

### 4.10 Lactate production assay

Total lactate content was measured from supernatant of 4-OI (100 μM) and LPS (100 ng/ml) treated, as well as *IRG1* siRNA transfected macrophages. Lactate was measured as described in Ref. [49].

### 4.11 Statistical analysis

Statistical analysis was done using Graphpad software. Two-sided paired Student's t-tests were applied to determine significance. For multiple comparisons, one-way ANOVA was applied depending on the experimental set-up. Significance level alpha threshold is indicated by a *P*-value below 0.05.

## Funding statement

This work was supported by the 10.13039/501100000781European Research Council (ERC) under the 10.13039/100010661European Union's Horizon 2020 research innovation programme [grant agreement No. 862137] to GvdB; 10.13039/501100001826ZonMW [project grant No. 09120011910001] to GvdB.

## Declaration of competing interest

All authors declare that they have no conflicts of interest.

## Data Availability

Data will be made available on request.
