# Supplementary material for: Itaconate promotes a wound resolving phenotype in pro-inflammatory macrophages
Source: Redox Biol. 2022 Dec 24;59:102591. doi: 10.1016/j.redox.2022.102591 (PMC9800195; doi:10.1016/j.redox.2022.102591)
Supplement: Multimedia component 1 [file mmc1.docx]

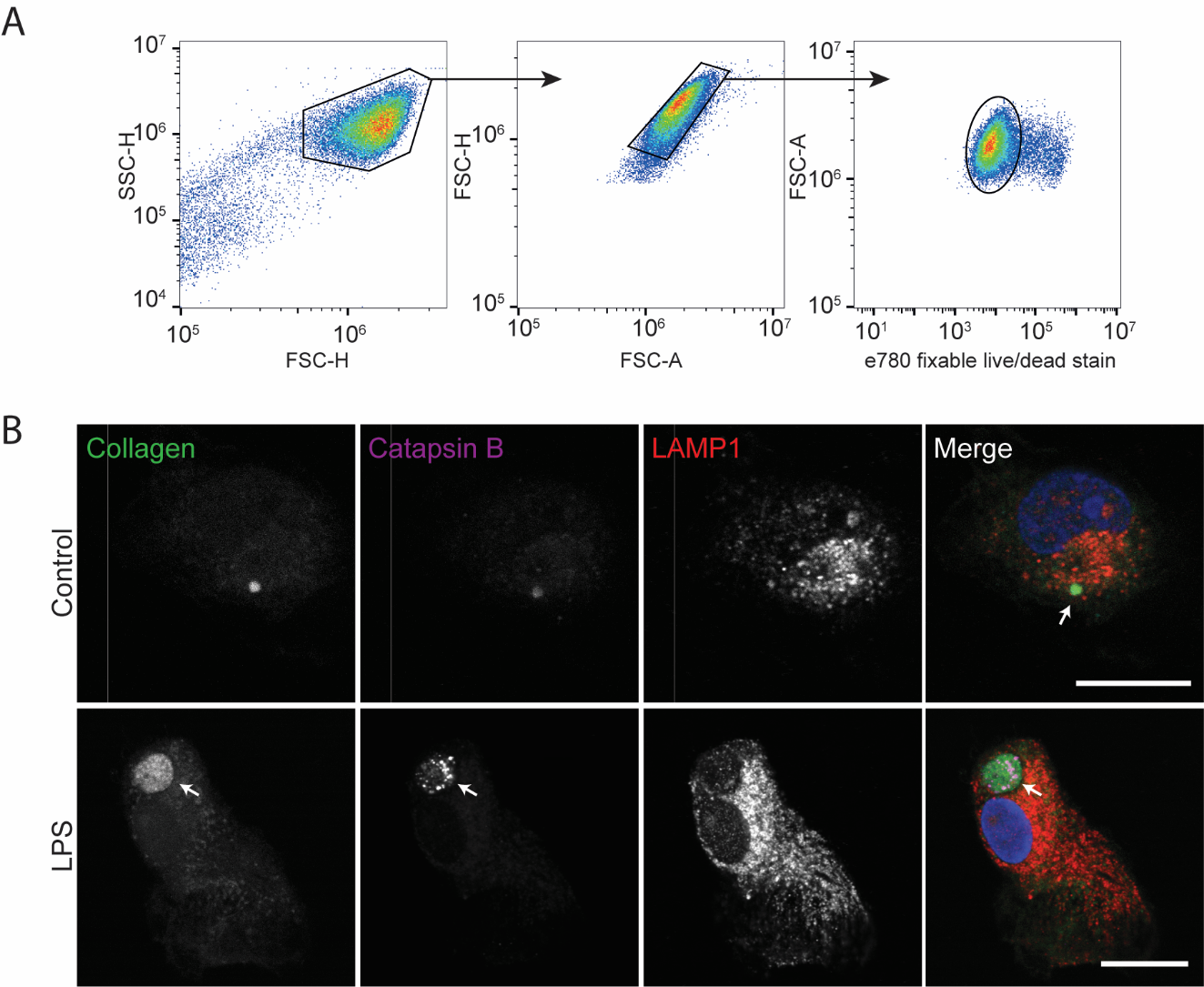


**Supplementary Figure 1. Pro-inflammatory human blood monocyte derived macrophages (hMDM) ingest collagen type I. A)** Flow cytometry gating strategy of macrophages after incubation on fibrous FITC-labelled collagen, detachment, staining with fixable e780 live/dead staining and PFA fixation. First, the cells were gated on forward scatter height (FCS-H) and side scatter height (SSC-H), followed by gating on FCS-H and FCS area (FCS-A) to remove doublets, and finally gated on e780 negative (living) cells. **B)** Representative confocal micrographs of macrophages treated with and without LPS and cultured on FITC-labelled collagen (green in merge). Cells were immunostained for lysosomal marker LAMP1 (red) and lysosomal protease cathepsin B (magenta). Arrows: intracellular compartments containing collagen-FITC. Scalebars are 10 µm.


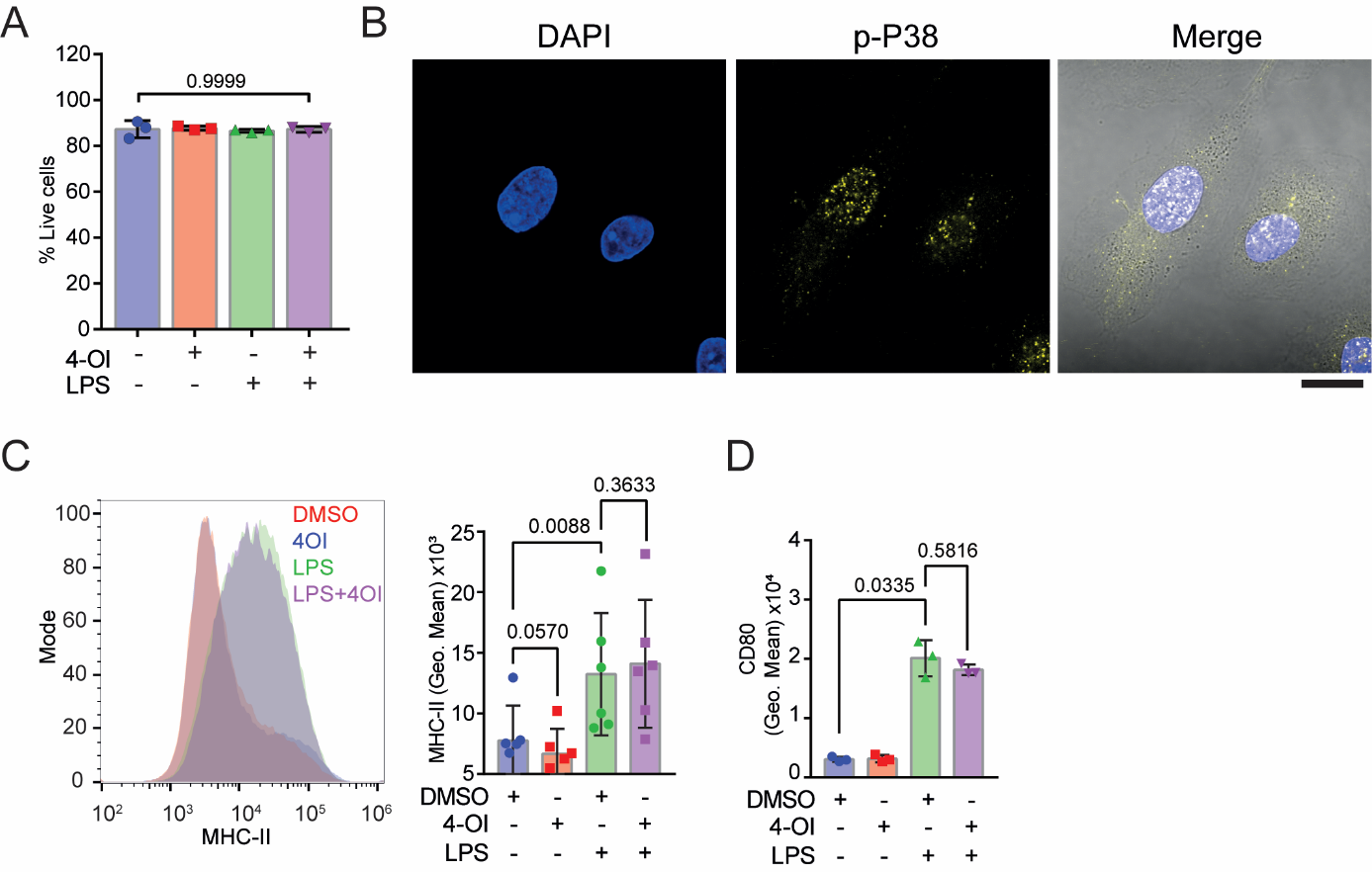


**Supplementary Figure 2. Inflammatory surface markers of human blood monocyte derived macrophages (hMDM) are not affected by 4-OI. A)** Percentages of e780 negative (i.e., viable) cells treated with 4-OI, LPS or both for 24 hrs by flow cytometry (*n*=3, one-way ANOVA with a Dunnett’s test for multiple comparison). **B)** Representative confocal micrographs of immunostained for phosphorylated-p38 MAPK (Thr180/Tyr182) (yellow) to validate nuclear localisation. Blue: DAPI. Scalebar is 10 µm. **C)** Representative flow cytometry histogram and quantification of MHC-II (HLA-DR) staining of cells treated with 4-OI, LPS or both for 24hrs (*n*=6, one-way ANOVA with a Tukey test for multiple comparison). **D)** Quantification of flow cytometry of CD80 staining of cells treated with 4-OI, LPS or both for 24hrs (*n*=6, one-way ANOVA with a Tukey test for multiple comparison). Data points show individual donors.


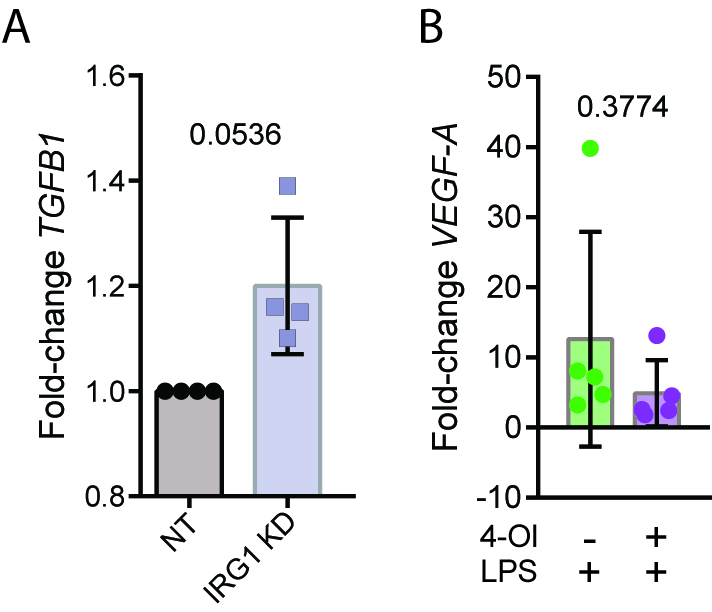


**Supplementary Figure 3. IRG1 knockdown non-significantly increased TGFB1 and 4-OI does not change VEGF-A expression in human blood monocyte derived macrophages (hMDM). A)** Fold-change mRNA of *TGFB1* by qPCR for macrophages with knockdown of IRG1 and treated with LPS as seen in main figure 2B. NT: non-targeting siRNA control. **B)** Vascular endothelial growth factor-A (*VEGF-A)* mRNA levels by qPCR of macrophages treated with LPS and with or without 4-OI for 24 hrs normalised against controls without LPS (*n*=5, paired t-test). Data points show individual donors.


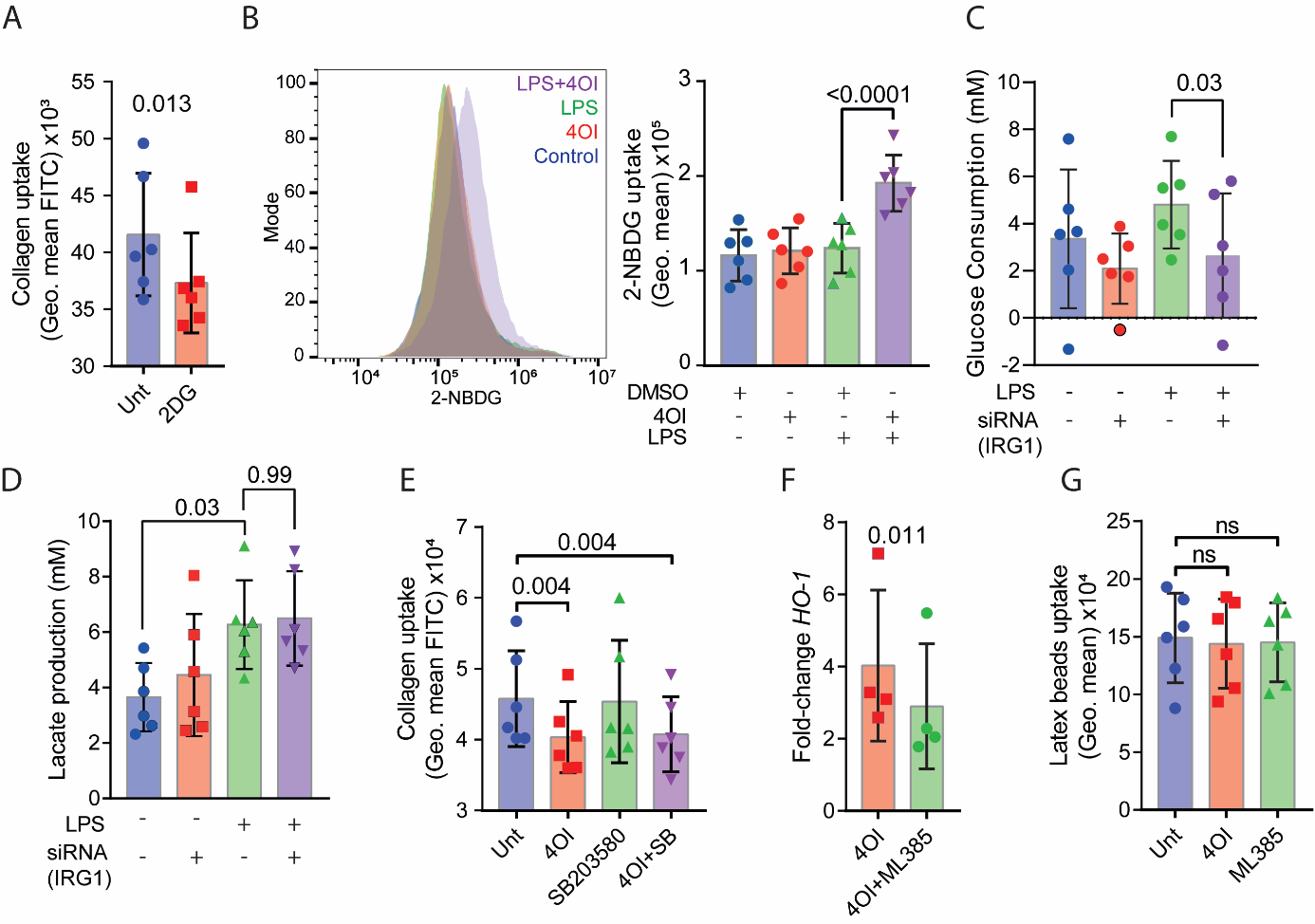


**Supplementary Figure 4. Control experiments with human blood monocyte derived macrophages (hMDM). A)** Collagen uptake experiment with and without glycolysis inhibitor 2-deoxy glucose (20 mM) for 24 hrs *(n*=6, paired t-test). Data points show individual donors. **B)** Representative flow cytometry histogram and quantification of uptake of fluorescent glucose analogue 2-NBDG by macrophages after 24 hrs incubation with LPS, 4-OI, or both as measured by geometric mean fluorescence intensity. DMSO: solvent control. (*n*=6 donors, one-way ANOVA with a Dunnette’s multiple comparison test). **C)** Glucose reduction of media in which IRG1 knockdown hMDMs were cultured treated with and without LPS. hMDM were either transfected with siRNA for the knockdown of IRG1 (+) or a control siRNA (-) (*n*=6 donors, paired t-test). **D)** Lactate production of media in which IRG1 knockdown hMDMs were cultured treated with and without LPS. hMDM were either transfected with siRNA for the knockdown of IRG1 (+) or a control siRNA (-) (*n*=6 donors, one-way ANOVA with a Tukey’s multiple comparison test). **E)** Collagen uptake experiment with unstimulated hMDMs treated with 4OI and p38 inhibitor SB208530 for 24 hrs. (*n*=6, one-way ANOVA with a Dunnett’s test for multiple comparison). **F)** Fold-change mRNA of heme oxygenase-1 (*HO-1*) by qPCR of macrophages treated with 4-OI, NRF2 inhibitor ML385 or both for 6 hrs (*n*=4, paired t-test). **G)** Uptake of latex beads for macrophage treated with 4-OI or ML385 for 24hrs (*n*=6, one-way ANOVA with a Dunnett’s test for multiple comparison). Data points show individual donors.
